# The complete mitochondrial genome of the yellow-spotted triggerfish (*Pseudobalistes fuscus*)

**DOI:** 10.1080/23802359.2016.1199002

**Published:** 2016-10-17

**Authors:** Kai Zhang, Xinxin You, Junmin Xu, Qiong Shi

**Affiliations:** a BGI Education Center, University of Chinese Academy of Sciences, Shenzhen, China;; b Shenzhen Key Lab of Marine Genomics, Guangdong Provincial Key Lab of Molecular Breeding in Marine Economic Animals, Shenzhen, China;; c Laboratory of Aquatic Bioinformatics, BGI-Zhenjiang Institute of Hydrobiology, Zhenjiang, China

**Keywords:** Balistidae, mitochondrial genome, *Pseudobalistes fuscus*

## Abstract

The yellow-spotted triggerfish (*Pseudobalistes fuscus*), a member of the genus *Pseudobalistes,* belongs to the family Balistidae. Here, we describe the complete mitochondrial genome sequence of *P. fuscus*. The genome, 16,480 bp in length, is comprised of 13 protein-coding genes, 22 tRNAs, 2 rRNAs and a major non-coding region. The gene content and order are in accord with the common vertebrate form. A phylogenic tree was constructed based on the complete mitogenomes of *P. fuscus* and six closely related species to estimate their phylogenic relationship. Our data present an important genetic resource for the biological studies of the family Balistidae.

The yellow-spotted triggerfish (*Pseudobalistes fuscus*) belongs to the family Balistidae. As a member of the genus *Pseudobalistes*, the fish has been distributed in Red Sea south to Durban, South Africa and east to the Society Islands, north to southern Japan, south to the southern Great Barrier Reef in Australia and New Caledonia. Mitochondrial DNA (mtDNA) is an important genetic marker and has been used for the identification of animal species. Mitochondrial genes have some obvious characteristics, such as a high mutation rate, lack of recombination and high intracellular copy numbers (Bruford et al. 2003; Armstrong & Ball 2005). In this study, for the first time, we obtained the complete mitochondrial genome sequence of *P. fuscus*.

The fish was collected from Sanya, China. Initially, the fish was identified based on both the morphological features and the *COX1* sequence. The specimen was stored at China National Genebank (Accession no. SY2014112514). Tissue samples were kept at 95% ethanol (Ruan et al. 2016). Total genomic DNAs were extracted from the fish fins using traditional phenol–chloroform extraction method (Taggart et al. 1992). A library of the whole genome with an insert size of 250 bp was prepared and sequenced on the Illumina HiSeq 4000 platform (San Diego, CA) at BGI-Shenzhen, China. A total of 2.5 Gb of raw reads were filtered with a Perl script to remove reads containing adaptor contamination. Subsequent *De novo* assemblies were generated by taking advantage of SOAP denovo-Trans (-K 71) (Tang et al. 2015).

The *P. fuscus* mitochondrial genome is 16,480 bp and has been submitted to NCBI (accession number: KU985150). We annotated the complete genome sequence using DOGMA (Wyman et al. 2004). It was found to be comprised of a non-coding control region and the classical 37 genes of vertebrate, including 13 protein-coding genes (PCGs), 22 transfer RNA genes, 2 ribosomal RNA genes and 1 putative control region. The order of all genes is identical to those in other typical vertebrate species. Twenty-two tRNAs stranded 20 kinds of amino acids, ranging in length from 67 to 75 bp. The length of 12s rRNA and 16s rRNA was 952 bp and 1675 bp, respectively. They were located between *tRNA^Phe^* and *tRNA^Leu^* genes and separated by the *tRNA^Val^* gene. Nucleotide base composition declines in the following order: C (29.21%)>A (29.13%)>T (25.70%)>G (15.96%).

To further validate the new sequence, we used mitochondrial genome sequences from six closely related species to construct a phylogenetic tree. We applied MEGA6 (Tempe, AZ; Tamura et al. 2013) to perform phylogenetic analysis using the maximum-likelihood method. The phylogenetic tree (Figure 1) clearly demonstrated the phylogenetic relationship of *P. fuscus* and its related species. We confirmed that *P. fuscus* was clustered with *Pseudobalistes flavimarginatus,* another previously reported triggerfish. These results provide a valuable resource for studies on the genetics and adaptive evolution of triggerfishes.

**Figure 1.**
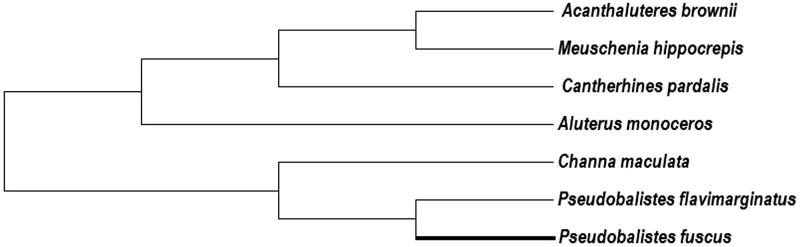
The phylogenetic tree of *P. fuscus* and six closely related species with reported mitogenome sequences. Genbank accession numbers: *Acanthaluteres brownie,* NC_011947.1; *Aluterus monoceros*, NC_027268.1; *Cantherhines pardalis*, NC_011325.1; *Channa maculata*, KC823606.1; *Meuschenia hippocrepis,* NC_011956.1*; Pseudobalistes flavimarginatus*, NC_011939.1; *Pseudobalistes fuscus*, KU985150. The bold branch highlighted the studied triggerfish in this article.
